# Supplementary material for: Association between the PPARGC1A Gly482Ser polymorphism and muscle fitness in Chinese schoolchildren
Source: PLoS One. 2023 Apr 27;18(4):e0284827. doi: 10.1371/journal.pone.0284827 (PMC10138255; doi:10.1371/journal.pone.0284827)
Supplement: Attachment — Submitted filename: Response to reviewer1.docx [file pone.0284827.s001.docx]

Reviewer #1: I congratulate the authors on the study.
However, there are some points to improve.
Abstract: completely revise it. Review the background regarding muscle development in children, specify the definition and problem correlation of the PPARGC1A gene. Enter the definition first and then the gene in parentheses. Also, improve the explanation of materials and methods, are unclear. Improve the difference between Gly/Gly and Gly/Ser before explaining the results.Improve explanation of findings and conclusion.

Response to reviewers: Due to the word limit of the abstract, the background of muscle development in schoolchildren is detailed in INTRODUCTION. The Gly482Ser polymorphism of the *PPARGC1A* gene includes two allele (Gly,Ser), which are divided into three genotypes Gly/Gly,Gly/Ser,Ser/Ser are described in the methods. Other changes have been colour coded in revision.

Introduction: Expand the background and bibliographic references regarding muscle development in children, the correlation with health and the possible negative consequences at a physiological and pathological level in case it is not corrected. Improving the binding to the gene and the respective polymorphisms and their consequences.
Improve exposure of the problem.

Response to reviewers: The background and bibliographic references haver been expanded and colour coded in revision.

Insert line 86 the various types of tests used for the evaluation of physical strength, after the explanation of the problem, increase the bibliographic references on the validity of the tests.
Response to reviewers: The bibliographic references on the validity of the tests have been in the introducion ,the references 10-17 are the latest studies and reviews assessing the validity of muscle assessment tests in children.
Materials and Methods: Write a paragraph on statistical analysis and how it was evaluated. A table could be inserted with the tests carried out, the repetitions, the modalities and the respective bibliographic references.

Response to reviewers: The statistical analysis and how it was evaluated were described in Line 149-154.The bibliographic references of the tests were marked.
Discussion: improve the exposition of the problem and the background as in the in**troduction with more in-depth analysis of the existing bibliography in the lit**erature. Also, improve exposure regarding the innovation that the study can bring to research. Improve the link between BMI and Gly482, increase bibliographic references and exposure of the link.

Response to reviewers: This study was aimed to investigate the association between genetic polymorphism and muscle in children. One of the innovations was that there were no previous genetic studies of the *PPARGC1A* polymorphism in children's muscle, only in athletes and adults, and the second innovation was to replace the invasive muscle fiber experiment with a muscle evaluation index. As the existing literature focuses on athletes and adults, there are few studies on genetic polymorphisms in children's muscles and the depth of our analysis is limited, and we will keep exploring this direction in the future with more in-depth analysis . The link between BMI and Gly482 were improved with bibliographic references in Line 286-290.
